# Incremental impact of community-delivered HPV self-sampling on screening uptake within an active outreach system: A quasi-experimental implementation study in rural Thailand

**DOI:** 10.1371/journal.pone.0349531

**Published:** 2026-06-01

**Authors:** Jiraporn Butta, Phanthitra Thonghoi, Tipamanee Kesakul, Amawasri Thoopthong, Wongsirirak Butdee, Somthawin Pirakittivorakul, Fuangfah Thongdara, Patchana Hengboriboonpong Jaidee, Manachai Yingklang

**Affiliations:** 1 Faculty of Public Health, Burapha University, Chonburi, Thailand; 2 Prokfa Sub-district Health Promotion Hospital, Chonburi, Thailand; Sefako Makgatho Health Sciences University, SOUTH AFRICA

## Abstract

**Background:**

Thailand has still not achieved the cervical cancer screening coverage target set by the World Health Organization (WHO). Although HPV self-sampling has been introduced to reduce access barriers, delivery remains largely facility-based, requiring women to attend primary care units during working hours. Community-delivered approaches have been proposed as an alternative; however, evidence comparing strategies within the same service context remains limited. This study aimed to assess the incremental benefit of a community-delivered approach, incorporating structured workshops and same-day sample return, within an already active outreach system, compared with a facility-based approach with similar outreach support, on screening uptake; secondary outcomes included operational feasibility and acceptability.

**Methods:**

We conducted a quasi-experimental comparative study in a rural subdistrict of Thailand. Eligible women aged 30–60 years were recruited from two villages and allocated at the village level to either a community-delivered approach (CD group) or a facility-based approach with active outreach (FB group). Screening uptake was defined as the return of a completed self-sampling kit and is presented using proportions and 95% confidence intervals. Feasibility and acceptability were also assessed descriptively using process indicators and participant-reported experiences.

**Results:**

A total of 108 participants were enrolled, with 106 included in the primary analysis. Screening uptake was 52% (28/54; 95% CI: 42% to 61%) in the CD group and 35% (18/52; 95% CI: 26% to 45%) in the FB group. The observed absolute difference was 17% (95% CI: −1% to 34%). All returned samples were adequate for HPV testing. Participant-reported confidence, usability, and overall experience with self-sampling were favorable and similar across groups.

**Conclusions:**

HPV self-sampling was operationally feasible and acceptable under both delivery approaches in this rural Thai setting. Screening uptake was numerically higher in the community-delivered group; however, the confidence interval included no difference, indicating uncertainty in the direction and magnitude of effect. These findings are suggestive but inconclusive evidence. Further adequately powered studies with rigorous implementation evaluation are needed, particularly to optimize service delivery, including increasing flexibility in scheduling, strengthening implementation fidelity, and evaluating workforce and cost implications, to better determine effectiveness and scalability.

**Trials Registration:** Thai Clinical Trials Registry (TCTR): TCTR20241231010.

## Introduction

Cervical cancer is a public health issue for women around the world, especially in low- and middle-income countries (LMICs) such as Thailand [1,2]. Persistent infection with high-risk human papillomavirus (HPV), especially types 16 and 18, is the primary cause of the disease [3]. Although effective vaccines and screening tools are available, Thailand has not yet achieved the World Health Organization’s (WHO) target screening coverage among eligible women [4]. Barriers such as time constraints, embarrassment, fear of diagnosis, low health literacy, and limited access to healthcare facilities contribute to suboptimal screening rates [5,6]. While health education campaigns have improved awareness [7–9], most screening still relies on Pap smears and clinic-based services.

HPV self-sampling has emerged as a promising and flexible alternative to clinic-based screening [10–12]. Several studies have demonstrated its accuracy and acceptability across diverse populations [13–18]. In Thailand, the National Health Security Office (NHSO) has introduced free self-sampling kits available to reduce financial barriers [19]. These kits can be obtained from primary care units (PCUs) under the guidance of healthcare providers. However, challenges such as uncertainty about how to perform self-collection and difficulties in accessing PCUs during working hours have limited the uptake of this service [20–22].

Various strategies have been explored to improve participation in HPV self-sampling [23–25]. In high-income countries, mail-based HPV self-sampling has improved screening uptake by allowing women to collect samples at home without visiting a clinic [26,27]. However, these approaches assume high postal reliability, digital access, and individual autonomy factors that may not align with the realities of LMICs or rural areas [23,28]. In addition, cultural norms, preferences for interpersonal support, and concerns about privacy in shared living environments may further limit the feasibility of mail-based delivery in these settings. These contextual challenges have prompted consideration of alternative delivery approaches that are more closely aligned with local community structures.

Community-based approaches have been increasingly considered in LMICs, often leveraging existing local care networks, including community health workers and village health volunteers (VHVs), to support service delivery within communities [29–32]. In Thailand, VHVs play an established role in health promotion and outreach across a wide range of communicable and non-communicable diseases and serve as trusted intermediaries between communities and the formal healthcare system [33]. However, their involvement in cervical cancer screening, particularly in HPV self-sampling, varies across local implementation practices and is not standardized nationwide. In many settings, their role remains focused on health education, invitation, and follow-up rather than direct participation in screening delivery. Within this context, access to HPV self-sampling in most local settings continues to rely primarily on facility-based services, requiring women to attend primary care units during clinic operating hours. As a result, structural constraints related to time, travel, and service organization may continue to influence participation, even in areas with active community engagement. This raises the question of whether adapting HPV self-sampling delivery to a community-based approach is operationally feasible within existing health systems.

At the same time, HPV self-sampling programs are often implemented alongside varying levels of outreach support, ranging from passive communication to more proactive strategies such as home visits and digital reminders. Consequently, observed differences in screening uptake may reflect not only the mode of delivery but also the broader organization of services and the intensity of outreach activities. However, evidence directly comparing community-delivered and facility-based HPV self-sampling within the same real-world rural service context, particularly where active outreach is already in place, remains limited.

Therefore, this study aimed to assess the incremental benefit of a community-delivered approach, incorporating structured workshops and same-day specimen return, within an already active outreach system, compared with a facility-based approach with similar outreach support, in terms of HPV self-sampling uptake. Secondary outcomes included operational feasibility and acceptability, evaluated via participant-reported confidence, usability, and overall experience.

## Materials and methods

### Study design and setting

This quasi-experimental comparative study was conducted in Koh Chan Subdistrict, Chonburi Province, Thailand, between January and April 2025. The study area is predominantly rural, with agriculture as the main livelihood, and is located approximately 60 km from the provincial capital (Mueang District, Chonburi Province). The population consists mainly of Thai nationals, with strong reliance on local community networks, including VHVs, who play a central role in public health outreach. The study site was purposively selected because of its historically low cervical cancer screening coverage. Allocation was conducted at the village level to minimize operational complexity and reduce disruption to routine healthcare services. The original study protocol (S1 File) and the TREND Statement Checklist (S2 File) are provided as supporting information.

### Deviations from the study protocol

The intervention was originally planned to span eight weeks. In practice, the active intervention components, including community workshops, kit distribution, and sample collection, were implemented during weeks 2–4. Weeks 5–7 primarily involved waiting for HPV test results from the referral hospital, with minimal reminder activities. This operational adjustment shortened the active intervention period to seven weeks but did not alter the intervention content, outcome measures, ethical safeguards, or analytic approach. The deviation was logistical in nature and did not affect the scientific integrity of the study.

### Sample-size calculation, sampling, and group allocation

Based on differences in screening uptake reported in a previous comparative study by Ibáñez et al. [32], the sample size was calculated assuming a two-sided α of 0.05 and 80% power to detect an absolute difference of 20% between groups, using the n4Studies sample size calculation program. An initial sample size of 98 participants (49 per group) was required, which was increased to 108 participants (54 per group) to account for an anticipated 10% loss to follow-up.

Two villages with similar demographic characteristics were selected and randomly allocated to study arms using a simple random process (lottery method) conducted by the research team prior to participant recruitment. Allocation was performed at the village level, such that all eligible participants within a given village were assigned to the same study arm. One village was assigned to the community-delivered group (CD group) and the other to the facility-based group (FB group), with both groups receiving active outreach support. This cluster-level allocation was used to minimize operational complexity and reduce the potential for contamination within communities. The two villages were located approximately 10 km apart.

### Participants and recruitment

Eligible participants were Thai women aged 30–60 years, in accordance with the screening age range recommended by the Department of Public Health of Thailand. Inclusion criteria required participants to have resided in the study area for at least one year, not have undergone cervical cancer screening within the past 5 years, not be currently pregnant, and to have no prior diagnosis of cervical cancer or history of total hysterectomy. Women with cognitive or physical impairments that could interfere with self-sampling were excluded.

Participants in both study arms received comparable levels of active outreach through home visits conducted by female VHVs and through digital communication using the LINE application during week 1. In some cases, VHVs also used village halls as community gathering points to disseminate information and distribute invitations. Interested participants were allowed to choose an appointment date for screening based on their availability. A reminder was sent via LINE and phone call one day prior to scheduled sessions, and participants received preparation guidelines.

### Intervention and comparator

#### Community-delivered with active outreach support.

Participants in this group were invited to attend community-based workshops held at local school halls within their village during weeks 2–4 by VHVs. All community-based sessions were primarily delivered by two female trained healthcare professionals. Prior to implementation, all facilitators received training and briefing on the study protocol and session procedures. Female VHVs (n = 6) supported participant recruitment, communication, and coordination but were not responsible for delivering the core intervention components. Sessions followed a predefined standardized structure that included (1) an educational video, (2) demonstration of HPV self-sampling using an anatomical model, (3) step-by-step explanation of kit use, and (4) an opportunity for participant questions and clarification. VHVs received orientation to ensure familiarity with the intervention process and to support coordination activities prior to study implementation. No formal fidelity monitoring (structured observation checklists, quantitative adherence measures, or competency assessments) was conducted. Descriptions of session delivery and duration (approximately 40 minutes) are based on protocol guidance and field implementation rather than systematically recorded metrics. After the session, participants received the Evalyn Brush HPV self-sampling kit (Rovers Medical Devices BV; distributed by the National Health Security Office). Kits were labeled with barcodes for tracking. Private spaces were provided for immediate onsite self-sampling, and samples were returned on the same day.

#### Facility-based delivery with active outreach support.

Participants in this group received personalized invitations from female VHVs (n = 8) of their village, as well as promotional messages via Facebook, LINE, and brochures, encouraging them to collect HPV self-sampling kits at PCUs. Instructions were provided using printed brochures and personalized guidance by healthcare professionals. Participants then self-collected samples at the PCUs within the specified time frame. This group represents a facility-based approach with active outreach support only in this study area rather than nationwide routine passive care.

### Procedures

Participants in both study arms were invited to select a convenient session within a predefined three-day window during weeks 2–4. Self-sampling and specimen return were completed on the same day of attendance rather than being subject to a fixed return deadline. Participants in the CD group were asked to return their kits on-site immediately after the education session (same day) within their village, while the FB group had to return the kits to the PCUs during clinic hours. This timeframe was based on logistical constraints related to specimen transport. After sample collection, participants completed a structured questionnaire assessing their experience. Samples were transported to a certified laboratory for HPV testing at Chonburi Cancer Hospital, and results were communicated approximately three weeks later (weeks 5–7).

### Outcome measures

The primary outcome was screening uptake, defined as the return of an HPV self-sampling kit with a completed sample. Secondary outcomes included operational feasibility and acceptability, assessed using a set of pragmatic sub-indicators. Operational feasibility was evaluated based on successful kit distribution and return within the study timeframe, as well as laboratory-confirmed sample adequacy. Sample quality was assessed by the diagnostic laboratory at Chonburi Cancer Hospital. Samples were considered adequate if sufficient cellular material was detected. High-risk HPV testing for 14 types was performed using reverse transcription polymerase chain reaction (RT-PCR) (Roche Diagnostics Limited, Thailand). Acceptability was assessed using a self-reported questionnaire. No predefined benchmarks were specified, consistent with the exploratory nature of the study. The questionnaire was used from a previously published instrument [32]. The questionnaire assessing participants’ perceptions of HPV self-sampling consisted of two sections. Section 1 included six items on participants’ demographic characteristics: age, educational level, marital status, religion, occupation, and history of prior cervical cancer screening. Section 2 comprised 18 items assessing participants’ perceptions and experiences following the use of the self-sampling kit. Item 1 was an open-ended question, items 2–7 and 9–18 were closed-ended questions with predefined response options. Item 8 assessed participants’ emotional responses after using the self-sampling kit and included 10 sub-items (comfort, calmness, normality, safety, privacy, shame, fear, anxiety, frustration, and nervousness) measured on a six-point rating scale (none, little, normal, quite, a lot, and I don’t know). The questionnaire was translated into Thai and back-translated to ensure linguistic and conceptual equivalence. All items were self-administered immediately after completion of the self-sampling procedure.

### Data analysis

Descriptive statistics were used to summarize participant characteristics and study outcomes. Categorical variables are presented as frequencies and percentages, while continuous variables are reported as means with standard deviations. Demographic characteristics between groups were compared using chi-square or Fisher’s exact tests, and continuous variables were compared using independent t-tests. Screening uptake is presented as proportions with 95% confidence intervals. Differences between groups are described using absolute differences and unadjusted odds ratio (OR) with 95% confidence intervals as supplementary effect size estimates. Given the quasi-experimental design and the small number of clusters, all analyses were interpreted descriptively rather than inferentially. No adjustment for clustering was performed due to the inclusion of only two clusters. Analyses were conducted on both a per-protocol and an intention-to-treat basis. Statistical analyses were performed using STATA version 10.1.

### Human ethics statement

This study was approved by the Institutional Review Board at Burapha University (IRB1-135/2567, No. HS092/2567, Date of Approval: 11 December 2024). This study was conducted in accordance with the Declaration of Helsinki Guidelines. Participants were recruited between January and April 2025, with the first enrollment on 25 January 2025 and the last subject visit on 04 April 2025. Written informed consent was obtained from all participants before conducting the study. No identified personal information of participants was found. Women were recruited in both settings, cervical cancer screening by HPV self-sampling was done, and kits were provided free of charge. Participants received a small token (soap and alcohol-based hand sanitizer) of appreciation to compensate for their time and any inconvenience associated with study participation; this compensation was non-coercive and approved by the ethics committee. Women who tested positive for high-risk HPV were referred to local hospitals for confirmatory cytology and appropriate follow-up care in accordance with national guidelines. Participants who tested negative for HPV infection in both the intervention and control groups were advised to screen again for the next five years. The authors confirm that all ongoing and related trials for this intervention are registered. This trial was registered with the Thai Clinical Trials Registry (TCTR) under the identifier TCTR20241231010.

## Results

### Participant flow

**Fig 1:**
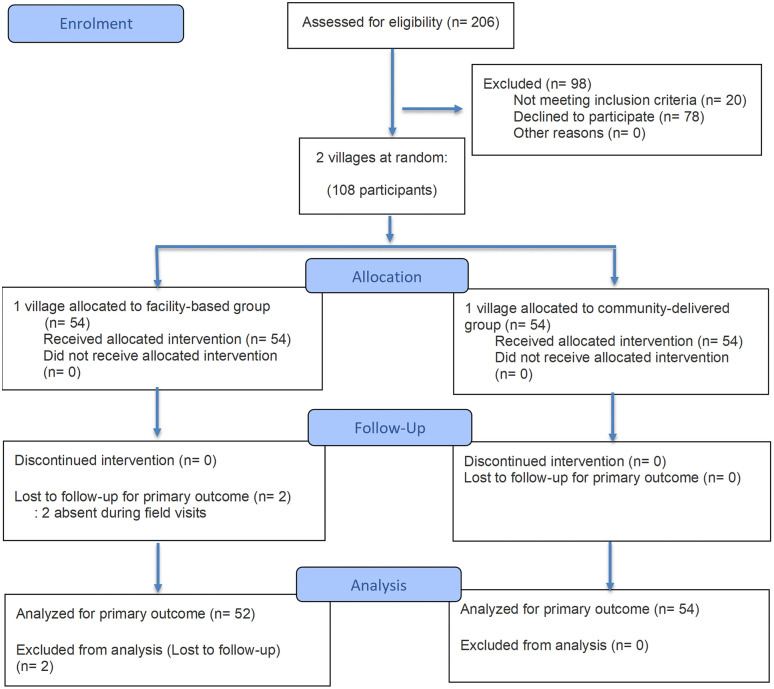
Flow diagram of participant flow in a quasi-experimental study.

Of 206 individuals assessed for eligibility, 98 were excluded (20 did not meet inclusion criteria and 78 declined participation). A total of 108 participants from two villages were allocated to the CD group (n = 54) or the FB group (n = 54). All participants received the allocated approach. During follow-up, two participants in the FB group were lost, while all participants in the CD group completed follow-up. Data from 54 participants in the CD group and 52 participants in the FB group were included in the primary analysis (Fig 1).

### Baseline demographics characteristics

Baseline characteristics were broadly comparable between the two groups (Table 1), with similar distributions observed across age, education level, occupation, marital status, religion, and prior cervical cancer screening history. Most participants had completed at least elementary education, and occupational backgrounds included agriculture, retail business, and office-based work. Similar proportions in both groups reported no previous cervical cancer screening.

**Table 1:** Baseline demographic characteristics of participants in the community-delivered and facility-based with active outreach groups.

Variables	Community-delivered with active outreach group (n = 54)		Facility-based with active outreach group (n = 54)		P-value*
	n	%	n	%	
**Age (years)**					
Mean ± SD	45.63 ± 8.48		47.19 ± 9.09		0.36^c^
**Education level**					
Illiterate	5	9	3	6	0.55^a^
Elementary	22	41	26	48	
Secondary 3	5	9	8	15	
Secondary 6/ vocational education	14	26	14	26	
Bachelor’s degree	7	13	3	6	
Higher than bachelor’s degree	1	2	0	0	
**Marital status**					
Single	9	17	10	19	0.71^a^
Married	36	67	35	65	
Divorce	8	15	8	15	
Other	1	2	1	2	
**Religion**					
Buddhism	54	100	53	98	0.31^b^
Islam	0	0	1	2	
**Occupation**					
Housewife	7	13	3	6	0.14^a^
Government official	1	2	1	2	
Agricultural	12	22	16	30	
Worker	10	19	21	39	
Retail business	13	24	8	15	
Officer	11	20	5	9	
**History of cervical cancer screening**					
Yes	19	35	17	32	0.79^a^
No	35	65	37	69	

^a^ *Based on the Chi-square test.*

^b^ *Based on the Fisher’s Exact test.*

^c^ *Based on the independent t test.*

### Screening uptake by delivery approach and HPV test results

In the primary per-protocol analysis, screening uptake was 52% (28/54; 95% CI: 42% to 61%) in the CD group and 35% (18/52; 95% CI: 26% to 45%) in the FB group (Table 2). The observed difference in screening uptake was 17%, with a 95% confidence interval of −1% to 34%, indicating uncertainty in both the direction and magnitude of effect. These results are suggestive but inconclusive. As a supplementary descriptive measure, the odds of screening uptake were numerically higher in the CD group than in the FB group (unadjusted OR = 2.03, 95% CI: 0.93 to 4.42). In a sensitivity analysis using an intention-to-treat approach, assuming participants lost to follow-up did not complete screening, uptake in the FB group was 33% (18/54), resulting in an absolute difference of 19% (95% CI: 0% to 37%). The corresponding unadjusted odds ratio was 2.15 (95% CI: 0.99 to 4.68). These findings were consistent in direction and magnitude with the primary analysis, although the confidence intervals indicate substantial uncertainty in the effect estimate.

**Table 2:** Unadjusted comparison of HPV self-sampling uptake between groups (per-protocol analysis).

Decision on cervical cancer screening using an HPV self-sampling kit	Community-delivered with active outreach group (n = 54)		Facility-based with active outreach group (n = 52)		Absolute difference	Unadjusted Odds ratio^a^
	n	%	n	%	% (95% CI)	OR (95% CI)
Yes	28	52	18	35	17% (−1% to 34%)	2.15 (0.99 to 4.68)
No	26	48	34	65		

^*a*^ *Based on the Chi-square test.*

A post-hoc power assessment was conducted based on the observed sample size and variance. Assuming a two-sided α of 0.05, the study had approximately 80% power to detect an absolute difference of about 27% between groups. The observed difference in screening uptake was 17%, which was smaller than this detectable threshold. This indicates that the study was underpowered to detect differences of this magnitude.

The most reported reason for non-participation in both groups was lack of time (CD group: 65%; FB group: 74%) (S3 File). All returned samples from both groups were adequate for HPV testing. Among valid samples (n = 46), five participants tested positive for high-risk HPV types other than HPV-16 and HPV-18.

### Participant-reported confidence and experience with self-sampling

Forty-six participants completed the self-sampling experience questionnaire (CD group: n = 28; FB group: n = 18). Overall ratings of the self-sampling experience were favorable in both groups. Most participants rated their experience as “good” or “very good” (79% vs. 83%) and reported that instructions were clear and easy to understand (71% vs. 89%) (S4 File). Self-reported confidence in having collected the sample correctly was similar between groups (79% in the CD group and 89% in the FB group). A higher proportion of participants in the FB group reported ease of brush insertion (89% vs.75%).

### Self-reported usability

Most participants described the self-sampling kit as easy to use (CD group: 75%; FB group: 94%), and more than three-quarters in both groups reported confidence in correct sample collection. Approximately half of participants reported no pain during sampling, while others reported mild discomfort, with similar distributions between groups.

**Fig 2:**
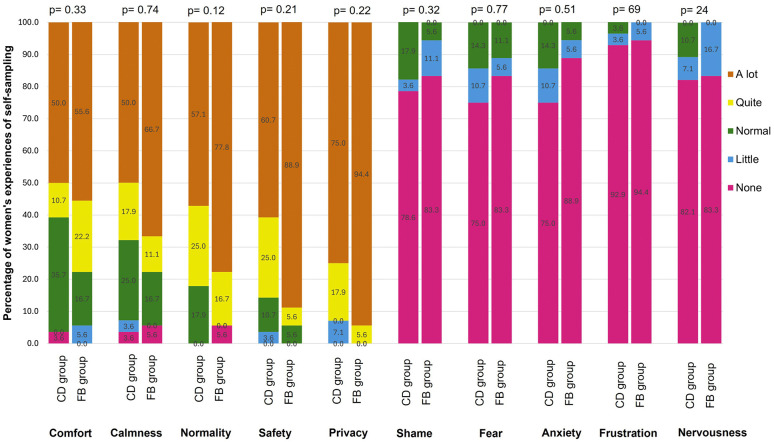
Perceptions of self-sampling use between study arms. Stacked bar charts show the percentage distribution of responses across experience domains. The community-delivered group (CD) received structured workshops and same-day return within an active outreach system, while the facility-based group (FB) received a facility-based approach with similar outreach support. P-values are based on the chi-square test comparing distributions between groups.

Emotional responses during self-sampling were predominantly positive across both groups (Fig 2). There were no differences between groups in reported comfort, calmness, normality, safety, privacy, shame, fear, anxiety, frustration, or nervousness (S4 File).

Time required to complete self-sampling was shorter in the FB group, with 94% completing the procedure within five minutes compared with 61% in the CD group. No serious adverse events were reported. Trust in test results and perceived health benefits were generally favorable in both groups, and all participants expressed willingness to recommend HPV self-sampling to others (S4 File).

## Discussion

This study examined whether adding community-delivered components to an already active outreach system could improve HPV self-sampling uptake in a rural Thai setting. Uptake was numerically higher in the CD group; however, the magnitude of the difference was modest, and the confidence interval included no difference. Results from both per-protocol and intention-to-treat analyses were consistent in direction. Statistical power should be considered when interpreting these findings. Based on the observed sample size and variance, the study had approximately 80% power to detect an absolute difference of about 27 percentage points between groups. The observed difference in screening uptake was 17%, which is below this detectable threshold, indicating that the study was underpowered to detect differences of this magnitude. These findings are suggestive but inconclusive and should not be interpreted as evidence of no effect.

Interpretation of these findings requires careful consideration of the implementation context. The facility-based group did not represent passive routine care but rather a facility-based approach with active outreach support. In this setting, proactive outreach was routinely delivered through established community health structures, including VHV-led invitations, digital communication, and personalized reminders. Such engagement may have enhanced motivation and emotional readiness, particularly among individuals initially hesitant to participate [24], and may have normalized self-sampling through interaction with trusted community actors [32,34–36]. This is consistent with evidence showing that proactive or opt-out invitation strategies can increase screening uptake [25]. Under these conditions, the observed difference is best interpreted as the incremental effect of adding community-delivered components within an already optimized outreach system, rather than a comparison with usual care.

Several design and implementation features likely attenuated the observed differences between groups. The use of proactive outreach in both study arms reduced between-group contrast and likely biased the effect estimate toward the null. Both study arms shared similar downstream processes, including specimen handling, transport logistics, and result communication pathways, and participation was organized through scheduled sessions within a predefined three-day window aligned with hospital-designated transport schedules. Although participants could select a convenient session and complete same-day sampling and return, these structural constraints may have limited the observable separation between delivery approaches. Furthermore, contamination between study arms is likely. Given the close geographic proximity and strong social interconnectedness of rural Thai communities, cross-village interaction through shared community spaces, kinship networks, and digital communication platforms was likely substantial. Even though sessions were conducted separately and HPV self-sampling is generally considered a private activity, such interaction could have reduced differences between groups.

HPV self-sampling was operationally feasible under both delivery approaches. Most participants successfully completed self-collection, and all returned samples were adequate for laboratory analysis. These findings are consistent with prior studies across diverse settings, including India [34,35], Kenya [37], Spain [32], and Sub-Saharan Africa [38], supporting the reliability of HPV self-sampling when appropriate support is provided. Despite overall feasibility, some differences in user experience were observed. Participants in the community-delivered group reported lower confidence in sample collection, potentially reflecting factors such as older age, limited familiarity with self-sampling procedures, or sexual inactivity. In contrast, participants in the facility-based group more frequently reported ease of brush insertion. However, these differences did not affect sample adequacy. These findings suggest that HPV self-sampling is generally acceptable and can be effectively performed across different delivery contexts. Additional supportive strategies, such as hands-on guidance or peer support, may further enhance user confidence.

In addition to feasibility, acceptability was high across both groups. This is broadly consistent with previous findings in Thailand, including a study by Noppawan Phoolcharoen et al. (2023), which reported acceptance rates exceeding 80% using a multidimensional definition of acceptability encompassing convenience, comfort, perceived safety, clarity of instructions, absence of pain or discomfort, and overall experience compared with clinician-collected smears [19]. Although measurement approaches and study contexts differed, these findings collectively suggest that HPV self-sampling is generally well accepted across both rural and urban Thai settings. However, these findings should be interpreted with caution. Acceptability was assessed using self-reported responses collected immediately after the activity in face-to-face community settings where healthcare personnel and VHVs were present. Although questionnaires were administered by study staff, full independence and strictly private conditions could not be ensured, which may have influenced responses. In rural Thai communities, VHVs are trusted figures, and participants may have been reluctant to express negative views or may have wished to show appreciation, introducing the possibility of social desirability bias. This concern is particularly relevant for the “willingness to recommend the kit” outcome, which was reported as 100% in both groups. Such uniformly positive responses are unlikely to reflect true universal agreement and are more plausibly explained by a ceiling effect combined with response bias. At the same time, the inherent convenience of HPV self-sampling may also have contributed to positive perceptions. Therefore, acceptability findings should be interpreted as upper-bound estimates rather than precise measures of user experience. Future studies should minimize this bias by employing more independent data collection approaches, such as anonymous or self-administered questionnaires, private response settings, or follow-up assessments conducted separately from the intervention context. Incorporating objective or mixed-method approaches may further strengthen the validity of acceptability evaluations.

Participants in both groups selected a convenient session within a predefined three-day window and completed self-sampling and specimen return on the same day. While this approach facilitated coordination of specimen transport and quality control, it may have imposed residual access constraints, particularly for working women or those with competing responsibilities. Consistent with this, lack of time was the most commonly reported reason for non-participation in both groups. These findings suggest that system-level constraints may have limited participation across both arms. Extending the service window or introducing more flexible return pathways such as additional sessions or decentralized drop-off options may increase overall uptake and potentially alter between-group differences by reducing shared barriers. This is supported by prior studies in Thailand using Pap smear screening [39,40] and in other countries using HPV self-sampling kits [34,41], which have shown improved uptake when more flexible service options are available. Providing extended return periods, community-based drop-off points, or home-return options via VHVs may further enhance accessibility while maintaining sample integrity.

From an implementation perspective, workforce considerations are critical for interpreting feasibility and scalability. In this study, core intervention components were delivered by trained healthcare personnel, while VHVs supported communication and participant engagement as part of their routine responsibilities. These activities were integrated within existing roles rather than requiring dedicated personnel or additional performance-based incentives. However, no formal assessment was conducted to quantify time requirements, workload distribution, or potential impacts on existing duties for either healthcare personnel or VHVs. Although the intervention was implemented within routine service structures, the absence of time–motion data or full-time equivalent (FTE) estimates limits the ability to determine whether these activities can be sustained at scale without affecting other responsibilities (e.g., community outreach, dengue surveillance, or maternal health services). Therefore, while the findings suggest that implementation within existing systems is feasible, the implications for workload balance, efficiency, and long-term sustainability remain uncertain. Future studies should incorporate explicit workforce and cost evaluations, including time requirements, task allocation, and opportunity costs, to better inform scalable implementation. These findings suggest that optimizing service delivery components, particularly flexibility in access and implementation fidelity, may be as important as the mode of delivery itself in improving screening uptake.

This study has several limitations. First, the quasi-experimental design without randomization limits causal inference, and residual confounding cannot be excluded. Second, cluster-level allocation was not accounted for in the analysis due to the small number of clusters, which may have led to underestimation of standard errors and overprecision of confidence intervals. Third, the study was conducted in a single subdistrict, which may limit generalizability. Fourth, the absence of formal fidelity monitoring precludes assessment of implementation consistency across sessions and limits interpretation of whether observed findings reflect true equivalence or variation in delivery quality. Fifth, contamination between villages cannot be excluded and may have biased the effect estimate toward the null. Sixth, feasibility was assessed descriptively without predefined operational benchmarks, limiting comparability with external standards. Seventh, acceptability outcomes were based on self-reported responses collected in face-to-face settings where VHVs were present, introducing potential social desirability and response bias, particularly for the uniformly high willingness-to-recommend outcome; the absence of blinding may have further contributed to bias. In addition, participants in both study arms received the same small, non-monetary token of appreciation (soap and alcohol-based hand sanitizer). Given its minimal value and equal provision across groups, this is unlikely to have introduced differential participation bias. Eight, specimen return was constrained by fixed transport schedules and service hours of subdistrict health-promoting hospitals, potentially limiting participation among working women. More flexible return pathways, such as extended return windows or decentralized drop-off points, should be evaluated in future implementation studies. Additionally, the study did not incorporate the role of family member involvement, which may be a key factor in supporting women’s participation in HPV self-sampling, particularly in close-knit rural communities. Finally, operational and scalability considerations were not fully assessed. Workforce requirements, time allocation, and cost-effectiveness were not quantified, and fixed service windows may have constrained participation. The modest sample size limited statistical power and precluded detailed inferential analyses. Future studies should incorporate rigorous implementation evaluation, including fidelity monitoring, workforce and cost assessment, and more flexible service delivery models.

## Conclusions

This study provides context-specific, implementation-relevant evidence that HPV self-sampling can be feasibly delivered through both community-delivered and facility-based approaches with active outreach in a rural Thai setting. The findings suggest that adding community-delivered components may provide incremental benefit; however, the evidence remains inconclusive. Further studies incorporating structured implementation evaluation, including fidelity monitoring, workforce assessment, larger sample sizes, and more flexible service delivery models, are required before conclusions regarding scalability and broader implementation can be drawn.

## Supporting information

S1 File The protocol for this trial. (PDF)

S2 File TREND statement checklist. (PDF)

S3 File Reasons for not participating in sample collection among non-participants. (DOCX)

S4 File Self-sampling experience between study arms (n = 46). (DOCX)

S5 File Dataset for analysis. (XLSX)
