# Supplementary material for: Incremental impact of community-delivered HPV self-sampling on screening uptake within an active outreach system: A quasi-experimental implementation study in rural Thailand
Source: PLoS One. 2026 Jun 1;21(6):e0349531. doi: 10.1371/journal.pone.0349531 (PMC13225424; doi:10.1371/journal.pone.0349531)
Supplement: S1 File — (PDF) [file pone.0349531.s001.pdf]

Research study protocol (English translation version)

1. Title

The Effect of Proactive Community Programs on Cervical Cancer Screening Uptake by Self-Collected Sampling

2. Principal Investigator

Dr. Manachai Yingklang

Faculty of Public Health, Burapha University

E-mail: manachai.yi@go.buu.ac.th

Phone: +66 93-329-4471

3. Co-Investigators

No.	Name	Student ID	Phone	E-mail
1	Ms. Jiraporn Butta	64070003	091-637-5892	64070003@go.buu.ac.th
2	Ms. Phanthitra Thonghoi	64070043	092-852-2639	64070043@go.buu.ac.th
3	Ms. Thipamanee Kesakul	64070086	098-796-1516	64070086@go.buu.ac.th
4	Ms. Amawasri Thoopthong	64070091	098-527-0098	64070091@go.buu.ac.th
5	Ms. Wongsirirak Butdee	64070101	064-456-0005	64070101@go.buu.ac.th

4. Background and Rationale

Cervical cancer remains a major global public health issue, including in every province of Thailand. In 2023, the incidence rate was 9,158 cases per 100,000 population, with a mortality rate of 4,705 per 100,000 population (HPV Information Centre, 2023). The primary cause of cervical cancer is infection with Human Papillomavirus (HPV), particularly types 16 and 18, along with other risk factors such as early sexual debut, multiple sexual partners, smoking, and immunosuppression (Okunade, 2020). Because cervical cancer is asymptomatic in its early stages, prevention relies heavily on HPV vaccination and regular screening to detect precancerous abnormalities (Viveros-Carreño et al., 2023).

Current screening methods include Pap smear cytology, HPV DNA testing, and Visual Inspection with Acetic Acid (VIA) (Bouvard et al., 2021). In some cases, two methods are combined to increase accuracy, such as Pap smear with HPV testing (Bedell et al., 2020). The World Health Organization (WHO) recommends screening women at ages 35 and 45, with at least 70% coverage (WHO, 2024). In Thailand, the Ministry of Public Health has set a

national target of screening at least 70% of women aged 30–60 years (Ministry of Public Health, 2024). Despite these recommendations, low screening participation remains a major obstacle, contributing to the persistently high incidence of cervical cancer.

Barriers to screening include lack of time, embarrassment during pelvic examinations, fear of test results, low awareness, limited knowledge about screening, financial constraints, poor accessibility to health services, religious beliefs, and lack of family or partner support (Gizaw et al., 2020). Dissatisfaction with health services, unfriendly staff, and long waiting times have also been shown to discourage women from attending screening (Atnafu et al., 2021). Studies in Thailand and other countries have developed health education programs and interventions to increase knowledge, improve attitudes, reduce anxiety, and overcome embarrassment associated with screening, which have effectively increased cervical cancer screening uptake (Abu et al., 2020; Ayanto et al., 2024; Bunkarn et al., 2020). However, most interventions still required women to undergo Pap smears performed by health professionals, which may not fully eliminate embarrassment as a barrier.

Recently, self-sampling for HPV testing has been promoted as an innovative alternative. This method allows women to collect vaginal samples themselves, reducing embarrassment. In Thailand, the National Health Security Office (NHSO) has distributed self-sampling kits free of charge to address financial barriers. Nevertheless, studies from other countries have found that women continue to express concerns regarding the accuracy and reliability of self-collected samples, as well as the inconvenience of collecting or picking up the kits (Gottschlich et al., 2019; Morgan et al., 2019). Additionally, information about self-sampling is not yet widely disseminated, leading to low confidence in the procedure. Therefore, building knowledge, understanding, and proper practices is a key strategy to overcome these barriers and improve uptake of self-collected HPV testing

From the literature review, only one study conducted in Thailand was identified that developed a health education program focused on providing knowledge for correct and high-quality self-sampling specimen collection for HPV testing, which yielded positive results (Sararut Chuleerat, 2024). However, that study did not evaluate the program's effectiveness in increasing cervical cancer screening uptake through self-sampling, nor did it address a major barrier: the lack of available time for participants to collect kits at health facilities. Research from Kenya suggested that proactive, community-based activities could enhance cervical cancer screening uptake via self-sampling (Huchko et al., 2018). Nevertheless, in Thailand, no study has demonstrated the effectiveness of community-based service delivery, as conventional approaches have relied primarily on facility-based invitations.

Therefore, this study aimed to examine the effect of a proactive community-based program on cervical cancer screening uptake through self-sampling among Thai women, using Koh Chan Subdistrict, Koh Chan District, Chonburi Province as a model area.

5. Objectives

1. To examine the effect of proactive community programs on cervical cancer screening uptake through self-collected sampling.
2. To evaluate the quality of self-collected samples obtained under the program.
3. To assess participants' opinions toward cervical cancer screening using self-collected sampling.

6. Hypotheses

H1: The proportion of women who undergo cervical cancer screening using self-collected sampling differs between the group receiving proactive community programs and the control group receiving the conventional program.

H2: The proportion of self-collected samples of adequate quality differs between the intervention group and the control group.

H3: The level of positive opinions toward cervical cancer screening by self-collected sampling will be high in both groups.

7. Research Questions

1. Is there a difference in the proportion of cervical cancer screening uptake using self-collected sampling between women who receive the proactive community program and those in the control group?
2. Is there a difference in the proportion of adequate-quality self-collected samples between the intervention and control groups?
3. What is the level of participants' opinions toward cervical cancer screening by self-collected sampling in both groups?

8. Expected Benefits

This study may serve as a model program to increase cervical cancer screening uptake in other communities.

9. Definitions

Cervical cancer screening: The collection of cervical cells from the vagina by the woman herself for HPV testing.

Intervention group: Women who receive the proactive community program developed in this study.

Control group: Women who receive the conventional cervical cancer screening promotion program.

Proactive community program: A community-based program consisting of educational activities about cervical cancer, HPV infection, and correct self-sampling procedures to ensure adequate quality for HPV testing. It also includes hands-on practice with mannequins to build confidence.

Conventional program: A program in which women are invited to pick up self-sampling kits at primary care units (PCUs) through online channels such as Facebook pages, LINE groups, and village health volunteers (VHVs). Staff provide instructions and distribute brochures for further self-study.

10. Literature Review and Related Studies

From the literature, several factors contribute to women's non-participation in cervical cancer screening. These include lack of time, embarrassment regarding pelvic examinations, fear of results, limited knowledge, low awareness of the importance of screening, financial barriers, limited access to health services, religious beliefs, and lack of support from family or partners (Gizaw et al., 2020). Dissatisfaction with health services or personnel, as well as long waiting times, can also deter women from screening (Atnafu et al., 2021). Addressing these barriers could substantially increase screening participation.

Research conducted in other countries and in various provinces of Thailand, such as Surat Thani Province, has demonstrated the effectiveness of health education programs and community-based interventions in improving knowledge, changing attitudes, reducing anxiety, and alleviating embarrassment. These interventions have successfully increased women's willingness to undergo cervical cancer screening (Abu et al., 2020; Ayanto et al., 2024; Bunkarn et al., 2020). However, most of these programs still relied on Pap smears performed by health professionals, leaving the barrier of embarrassment partially unresolved.

Recently, self-sampling for HPV testing has emerged as an innovative and widely accepted approach in many countries (Khoo et al., 2021; Nelson et al., 2017; Viviano et al., 2017), including Thailand (Phoolcharoen et al., 2018; Sangrajrang et al., 2023). Evidence

indicates that self-collected vaginal samples are comparable in quality to clinician-collected Pap smears (Aimagambetova et al., 2024). Furthermore, urine-based HPV testing, a newer method, has demonstrated high sensitivity (Nilyanimit et al., 2024). A study in Brazil reported high participation rates among women offered different methods: 100% returned urine samples, 93.8% returned self-collected vaginal samples, 90% participated in mobile Pap smear services, and 53.3% underwent hospital-based Pap smears (Lordelo et al., 2024), highlighting the strong potential of self-sampling methods.

Despite advantages such as privacy, convenience, and ease of use, self-sampling still faces barriers, including concerns about proper technique, lack of confidence in the results, and limited awareness of the method (Morgan et al., 2019; Song et al., 2024). In Thailand, a survey revealed that 33.6% of women still preferred clinician-collected Pap smears due to low confidence in self-sampling (Ploysawang et al., 2023). Therefore, building knowledge, understanding, and correct practice is critical to overcoming these barriers (Vega-Crespo et al., 2024).

Program development promoting self-sampling in Thailand remains limited. Only one study has created a health education program focused on correct self-sample collection for HPV testing, which improved sample quality (Sararut Chuleerat, 2024). However, this study did not evaluate the program's effectiveness in increasing screening uptake nor address barriers such as lack of time to pick up kits at health facilities. Evidence from other studies suggests that providing free self-sampling kits can remove financial barriers and significantly increase uptake (Hansen et al., 2024; Tranberg et al., 2018). The National Health Security Office (NHSO) of Thailand has already implemented this approach. Research in Kenya demonstrated that proactive community-based activities could further increase cervical cancer screening uptake using self-sampling (Huchko et al., 2018). In Spain, on-site training at health facilities improved screening rates (Ibáñez et al., 2023), while in the United States, mailing invitations and kits has proven effective (Winer et al., 2023). Nevertheless, the effectiveness of each strategy depends on local context; in developing countries, mail-based approaches may be less feasible due to infrastructural limitations (Table 1).

Table 1. summarizes international and Thai studies related to the development of programs and use of self-sampling methods for cervical cancer screening.

Country	Intervention/Activities	Outcomes	Recommendations/Benefits
**Norway** (Hansen et al., 2024)	3 groups:<br>- Group 1: Reminder to attend conventional screening<br>- Group 2: Option to order self-sampling kits online<br>- Group 3: Free self-sampling kits mailed to all participants	The free-kit group had the highest screening rate (27.7%) compared to other groups with lower participation	Providing free self-sampling kits increases screening uptake
**France** (Boyard et al., 2022)	Compared two approaches:<br>- Reminder to attend screening with a doctor<br>- Mail invitation to collect self-sampling kits	Mailing self-sampling kits yielded higher screening participation than reminders for physician-based screening	Mailing invitations for self-sampling is more effective
**USA** (Calderón-Mora et al., 2023)	Used video and PowerPoint presentations for health education	No significant difference in screening uptake between video vs. PowerPoint	Multimedia education helps improve understanding of content
**Brazil** (Lordelo et al., 2024)	5 groups:<br>- Group 1: Pap smear at hospital<br>- Group 2: Pap smear via mobile clinic<br>- Group 3: Urine-based HPV self-sampling<br>- Group 4: Vaginal HPV self-sampling<br>- Group 5: Participant's choice among methods	Screening participation:<br>- Group 1: 53.3%<br>- Group 2: 90%<br>- Group 3: 100%<br>- Group 4: 93.8%<br>- Group 5: 84.6%	1. Community/mobile services increase screening uptake 2. Women with prior Pap smears may hesitate to adopt self-sampling due to lack of confidence
**USA** (Winer et al., 2023)	2 groups: - Education group: Standard services + educational materials - Direct-mail group: Standard services + mailed self-sampling kits	Direct-mail group had higher return of self-sampling kits (61.7%)	Mailing self-sampling kits can substantially increase uptake
**Thailand (Udon)** (Sararut Chuleerat, 2024)	Phase 1: Interview to identify errors in self-sampling<br>Phase 2: Develop health education program<br>Phase 3: Evaluate knowledge and sample quality after program	Quality errors in self-collected samples decreased after program implementation	1. Program effectiveness depends on age, marital status, education level, and pre-existing attitudes 2. Partner/family involvement is important for social support and decision-making

HPV Self-Sampling Kit

The self-sampling kit used in this study was provided by the National Health Security Office (NHSO) through Ban Prokfa Primary Care Unit (PCU), in collaboration with Roche Diagnostics (Thailand) Co., Ltd., the National Cancer Institute, and Chonburi Cancer Hospital.

Instructions for Use of HPV self-sampling kit

1. Wash hands thoroughly with soap and dry them. Position yourself appropriately, such as sitting on the toilet or standing with one foot placed on the toilet seat.
2. Loosen the cap attached to the swab stick and gently pull it out only when ready to use. Do not touch the tip of the swab, and ensure that the swab head does not come into contact with any surface to avoid contamination.
3. Hold the swab handle at the marked area using two fingers.
4. Use your fingers to gently open the vaginal entrance and carefully insert the swab into the vagina until the marked fingers touch the vaginal opening.
5. Rotate the swab in one direction for about 10–30 seconds to collect the sample. Then, gently remove it.
6. Place the swab into the sample collection tube and close the cap securely. Return the tube to the staff as instructed.

Preparation guidelines for participants before the appointment for sample collection

- Do not undergo a pelvic examination within 24 hours before collection, as this may cause contamination.
- Collect the sample in the morning before bathing. Avoid vaginal douching with water or any liquid before collection.
- Do not wash or clean inside the vagina within 48 hours before the test, as it may remove cells needed for analysis.
- Abstain from sexual intercourse for at least 48 hours before the test.
- Collect the sample before menstruation or at least 7 days after menstruation ends.
- Eating and drinking are permitted as usual before screening.

11. Conceptual Framework

Based on the literature review, we developed a proactive community program to promote cervical cancer screening through self-sampling. The program focused on community-based activities, providing knowledge about cervical cancer, HPV infection, and correct self-sampling techniques to ensure adequate sample quality for HPV testing. It also included mannequin-based training to practice the use of self-sampling kits. The design was guided by COM_B model and Richard E. Mayer's Cognitive Theory of Multimedia Learning and John Dewey's Learning by Doing, which suggest that multimedia learning is more effective than relying solely on textbooks or written materials, as it fosters deeper and more comprehensive understanding. Accordingly, the program emphasized sustainable learning through hands-on practice. In addition, it explained test results and follow-up procedures in the case of positive findings, distributed self-sampling kits directly within the community to reduce time and travel barriers, and scheduled dates for sample submission to minimize forgetfulness. Participants' opinions on self-sampling were also assessed.

Independent Variable

Proactive community program consisting of

1. Public invitation to collect self-sampling kits at their village on specific dates and times.
2. Community activities including health education, mannequin-based demonstrations, hands-on practice, explanation of test results and follow-up procedures, and distribution of self-sampling kits.
3. Sending reminders and conducting follow-ups via LINE and phone calls prior to scheduled appointment dates.

Dependent Variables

Primary outcomes

1. Percentage of cervical cancer screening uptake through self-sampling (HPV testing).

Secondary outcomes

1. Quality of self-collected samples
2. Participants' opinions on self-sampling

12. Research type

This study employs a quasi-experimental design with a control group.

13. Research Ethics

Ethical approval will be obtained from the Burapha University Human Research Ethics Committee prior to data collection. Informed consent will be obtained from all participants before commencing the study.

14. Population and Sample

Population: Thai women aged 30–60 years.

Sample: Thai women aged 30–60 years living in Koh Chan Subdistrict, Koh Chan District, Chonburi Province.

The sample size was 108 participants, calculated using the n4studies program with 95% confidence interval (Z = 1.96), Power = 80%, and expected difference of 20% in screening uptake between intervention and control (based on Ibáñez et al., 2023).

$$n_{trt} = \left[\frac{z_{1-\frac{\alpha}{2}} \sqrt{\bar{p}\bar{q}\left(1+\frac{1}{r}\right)} + z_{1-\beta} \sqrt{p_1 q_1 + \frac{p_2 q_2}{r}}}{\Delta} \right]^2$$

$$p_1 = P(outcome|treatment), q_1 = 1 - p_1$$

$$p_2 = P(outcome|control), q_2 = 1 - p_2$$

$$\bar{p} = \frac{p_1 + p_2 r}{1+r}, \bar{q} = 1 - \bar{p}, r = \frac{n_{con}}{n_{trt}}$$

Z(0.975) = 1.96,

Z(0.8) = 0.84,

Proportion in group1 (p1) = 0.75,

Proportion in group2 (p2) = 0.95,

Ratio (r) = 1.0,

n1 = 48.79,

Thus, sample size for group1 = 49, and group2 = 49

From the calculation, 49 participants per group were required. To account for a 10% drop-out rate, the final sample size was set at 54 participants per group, totaling 108 participants.

15. Sampling Method

The study site was selected as Koh Chan Subdistrict, Koh Chan District, Chonburi Province, due to convenience in data collection and local cooperation. However, according to the 2024 HDC database, the cervical cancer screening rate in this area did not meet the national target of 60%, reflecting the same issue seen nationwide. Two villages were randomly selected using simple random sampling (lottery method).

Village 3 (Ban Prong Hin): Intervention group

Village 5 (Ban Sra Taprom): Control group

The two villages are approximately 10 km apart to prevent information contamination. Recruitment was done by VHVs through posters and leaflets. Interested women were listed, and participants were randomly assigned, ensuring no significant differences between groups in terms of age, education, marital status, or prior cervical cancer screening.

Inclusion Criteria

1. Thai women aged 30–60 years
2. Not pregnant
3. Resided in Koh Chan Subdistrict for at least one year
4. No history of hysterectomy
5. No history or diagnosis of cervical cancer
6. Never previously undergone cervical cancer screening using self-sampling
7. If previously screened by Pap smear, it must be within the scheduled follow-up year
8. Able to read and speak Thai
9. Willing to participate in the study

Exclusion Criteria

1. Withdrawal of consent to participate

16. Experimental Design

Two groups were assigned: the intervention group (proactive community program) and the control group (conventional program). The intervention period for participants will last 2 months, with the total project duration being 4 months.

17. Access to Participants

Prior to initiating the study, we coordinated with the director of Ban Prok Fa Health Promoting Hospital, Koh Chan Subdistrict, Koh Chan District, Chonburi Province, as well as with community leaders and village health volunteers (VHVs), to obtain permission to conduct the research. Once approval was granted, information about the study was disseminated, and potential participants were invited through VHVs, posters and leaflets. Interested individuals were listed and randomly assigned. The researcher then explained the study procedures and distributed informed consent forms at the village hall, asking participants to complete them at home. A date and time were scheduled for collecting the signed consent forms. Data were collected using research record forms and questionnaires. All information was treated as strictly confidential and used solely for research purposes, with no disclosure of personal data under any circumstances. Confidentiality was maintained for one year, during which data were stored as encrypted electronic files accessible only to the researcher. Upon completion of the study, all data were permanently destroyed.

18. Research Procedures (8 weeks)

Week 1: Recruitment, Consent, and Invitation

Coordinated with the director of Ban Prok Fa Health Promoting Hospital, Koh Chan Subdistrict, Koh Chan District, Chonburi Province, and with community leaders to obtain permission to conduct the study, as well as disseminated recruitment information through posters.

- Scheduled meetings with participants to provide information and obtain written informed consent. Participants were assigned into intervention and control groups, and the consent meetings were held on different dates, times, and locations for each group. Participants were allowed to choose an appointment date for screening based on their availability.

Week 2: Intervention

Before initiating the activities, the researcher provided education and counseling on cervical cancer screening through self-sampling, interpretation of test results, and the referral process for treatment.

Intervention group

Participants attended a single 40-minute session designed to promote self-sampling. The session included health education and hands-on training using a mannequin to practice

specimen collection. Throughout the activity, all research assistants were required to wear masks at all times, and alcohol-based hand sanitizers were provided to participants before and after the session as a precaution against COVID-19 transmission.

Control group

Participants received cervical cancer screening through self-sampling using the routine promotion approach currently implemented by Ban Prok Fa Health Promoting Hospital. To minimize deviations from routine practice due to awareness of the research, the researcher explained the study and requested staff to continue providing services strictly according to the usual procedures to ensure validity of the study findings.

Prior to specimen collection, participants in both groups were given instructions regarding the appropriate timing and precautions, as follows:

- No pelvic examination within the past 24 hours (to avoid contamination from medications or lubricants)
- Preferably collect samples in the morning before bathing, avoiding douching with water or any liquid prior to sampling
- No vaginal washing or cleansing within 48 hours before the test (to ensure sufficient cell collection)
- Abstain from sexual intercourse for at least 48 hours before the test
- Collect specimens before menstruation or at least 7 days after menstruation
- Eating and drinking were permitted as usual

In addition, the health facility provided leaflets with instructions on how to collect the self-sampling specimens.

Table 2. Comparison of procedures between intervention and control groups.

Intervention Group (Proactive Program)	Duration	Control Group (Routine Program)
1. Publicized the self-sampling kit distribution at the village hall on the scheduled date and time.	–	1. Publicized the self-sampling kit distribution at Ban Prok Fa Health Promoting Hospital via online channels (LINE) and through village health volunteers. Participants could choose a convenient time and either collect the specimen at home or at the health facility. Kits were provided once.
2. Conducted a community-based activity including: - Education on HPV and cervical cancer - Explanation of result interpretation and referral for follow-up care if positive - Demonstration of self-sampling using a mannequin - Hands-on practice using the	40 minutes	2. Female staff at Ban Prok Fa Health Promoting Hospital distributed self-sampling kits, explained specimen collection, and provided leaflets for self-study. Participants collected specimens at the facility and completed questionnaires.

Intervention Group (Proactive Program)	Duration	Control Group (Routine Program)
mannequin - Distribution of self-sampling kits and post-use feedback questionnaires.		
3. Distributed post-use feedback questionnaires; researcher followed up one day prior to scheduled collection via phone or LINE.	–	3. Follow-up reminders via phone or LINE one day prior to scheduled collection.

Weeks 3–5: Sample Collection

- For the intervention group, the researcher visited the community at scheduled times to directly collect the self-sampling specimens and the post-use feedback questionnaires. In addition, the research team followed up with participants by phone or LINE messages one day in advance to inquire about any difficulties in specimen collection and to remind them to return the samples on time.
- For the control group, participants were instructed to return their self-sampling specimens and feedback questionnaires in person at Ban Prok Fa Health Promoting Hospital within two weeks after receiving the kits. If specimens had not been returned within the specified timeframe, the research team conducted follow-up reminders via phone or LINE one day before the due date.

Weeks 6–8: Test Results and Follow-Up

- Laboratory testing, specimens were sent to the Chonburi Cancer Hospital laboratory for HPV analysis. Results were expected within 7–14 days, and testing was provided free of charge; any costs incurred were covered by the researcher.
- Result notification and follow-up, given the sensitive nature of HPV testing among women, the researcher delivered results personally in sealed envelopes to participants' homes to maintain confidentiality and minimize potential distress. Participants received counseling and referral for further care if any risk was detected.
- Data analysis, outcomes related to screening uptake, specimen quality, and participant feedback were analyzed and summarized.

19. Research Instruments

- HPV self-sampling kits were provided by NHSO via Ban Prokfa PCU.
- Program manual – activities and self-sampling instructions.

Data collection tools

- Record forms for returned samples and quality evaluation (valid/invalid).
- Structured questionnaire with two parts. The self-sampling feedback questionnaire consisted of two sections. Section 1 collected demographic information from participants (6 items), including age, education level, marital status, religion, occupation, and history of cervical cancer screening. Section 2 assessed participants' opinions after using the self-sampling kit (18 items). Item 1 was an open-ended question, items 2–7 and 9–18 were close-ended multiple-choice questions, and item 8 evaluated participants' feelings after using the kit through 11 sub-items rated on a 6-point Likert scale. The questionnaire was adapted from a previously published study (Ibáñez et al., 2023) and was not newly developed in this study.

20. Outcomes

Primary outcomes

- Screening uptake measured by proportion of women who returned self-collected samples.

Secondary outcomes

- Sample quality categorized as valid/invalid based on RT-PCR results from the hospital.
- Opinions measured by structured questionnaire.

21. Statistical Analysis

After data collection, the researcher verified the completeness and accuracy of all data before proceeding with analysis according to the study objectives:

- Descriptive statistics were used to summarize the baseline characteristics of participants in the intervention and control groups.
- The Chi-square goodness-of-fit test was applied to compare general baseline characteristics between groups prior to the intervention, with a significance level of 0.05 and a 95% confidence interval.

- Cervical cancer screening uptake and specimen quality in both groups were presented as counts and percentages.
- Differences in screening uptake and specimen quality between the intervention and control groups were analyzed using the Chi-square goodness-of-fit test.

All data were anonymized, stored confidentially, and used solely for research purposes. No personal information of participants was disclosed under any circumstances.

22. Timeline

The study was expected to collect data from participants over approximately 2 months following approval by the Human Research Ethics Committee. The total duration of the project, from initiation to completion, was anticipated to be 4 months, from January to April 2025.

23. Budget

Total research budget: 50,000 THB

24. References

Abu, S. H., Woldehanna, B. T., Nida, E. T., Tilahun, A. W., Gebremariam, M. Y., & Sisay, M. M. (2020). The role of health education on cervical cancer screening uptake at selected health centers in Addis Ababa. *PLOS ONE*, 15(10), e0239580. https://doi.org/10.1371/journal.pone.0239580

Aimagambetova, G., Atageldiyeva, K., Marat, A., Suleimenova, A., Issa, T., Raman, S., Huang, T., Ashimkhanova, A., Aron, S., Dongo, A., Iztleuov, Y., Shamkeeva, S., & Azizan, A. (2024). Comparison of diagnostic accuracy and acceptability of self-sampling devices for human Papillomavirus detection: A systematic review. *Prev Med Rep*, 38, 102590. https://doi.org/10.1016/j.pmedr.2024.102590

Atnafu, T., Daka, D. W., Debela, T. F., & Ergiba, M. S. (2021). Women's Satisfaction with Cervical Cancer Screening Services and Associated Factors in Maternal Health Clinics of Jimma Town Public Health Facilities, Southwest Ethiopia. *Cancer Manag Res*, 13, 7685-7696. https://doi.org/10.2147/cmar.S327369

Ayanto, S. Y., Belachew, T., & Wordofa, M. A. (2024). Effectiveness of couple education and counseling on uptake of cervical cancer screening among women in Southern

Ethiopia: a cluster randomized trial. *Scientific Reports*, 14(1), 12557. https://doi.org/10.1038/s41598-024-61988-2

Bedell, S. L., Goldstein, L. S., Goldstein, A. R., & Goldstein, A. T. (2020). Cervical Cancer Screening: Past, Present, and Future. *Sex Med Rev*, 8(1), 28-37. https://doi.org/10.1016/j.sxmr.2019.09.005

Bouvard, V., Wentzensen, N., Mackie, A., Berkhof, J., Brotherton, J., Giorgi-Rossi, P., Kupets, R., Smith, R., Arrossi, S., Bendahhou, K., Canfell, K., Chirenje, Z. M., Chung, M. H., Del Pino, M., de Sanjosé, S., Elfström, M., Franco, E. L., Hamashima, C., Hamers, F. F., . . . Lauby-Secretan, B. (2021). The IARC Perspective on Cervical Cancer Screening. *N Engl J Med*, 385(20), 1908-1918. https://doi.org/10.1056/NEJMsr2030640

Boyard, J., Caille, A., Brunet-Houdard, S., Sengchanh-Vidal, S., Giraudeau, B., Marret, H., Rolland-Lozachmeur, G., Rusch, E., Gaudy-Graffin, C., & Haguenoer, K. (2022). A Home-Mailed Versus General Practitioner-Delivered Vaginal Self-Sampling Kit for Cervical Cancer Screening: A Cluster Randomized Controlled Trial with a Cost-Effectiveness Analysis. *Journal of Women's Health*, 31(10), 1472-1480. https://doi.org/10.1089/jwh.2021.0597

Bunkarn, O., Kusol, K., & Eksirinimit, T. (2020). The Outcome of a Self-Efficacy Enhancement Program for Cervical Cancer Screening among Women in Phrasaeng District, Suratthani Province, Thailand. *Asian Pac J Cancer Prev*, 21(7), 2075-2081. https://doi.org/10.31557/apjcp.2020.21.7.2075

Calderón-Mora, J., Alomari, A., & Shokar, N. (2023). Comparison of Narrative Video and Flipchart Presentation to Promote Cervical Cancer Screening Among Latinas Along the Border. *Health Education & Behavior*, 50(5), 561-571. https://doi.org/10.1177/10901981221074918

Gizaw, M., Teka, B., Ruddies, F., Kassahun, K., Worku, D., Worku, A., Wienke, A., Mikolajczyk, R., Jemal, A., Kaufmann, A. M., Abebe, T., Addissie, A., & Kantelhardt, E. J. (2020). Reasons for Not Attending Cervical Cancer Screening and Associated Factors in Rural Ethiopia. *Cancer Prev Res (Phila)*, 13(7), 593-600. https://doi.org/10.1158/1940-6207.Capr-19-0485

Gottschlich, A., Nuntadusit, T., Zarins, K. R., Hada, M., Chooson, N., Bilheem, S., Navakanitworakul, R., Nittayaboon, K., Virani, S., Rozek, L., Sriplung, H., & Meza, R. (2019). Barriers to cervical cancer screening and acceptability of HPV self-testing:

a cross-sectional comparison between ethnic groups in Southern Thailand. *BMJ Open*, 9(11), e031957. https://doi.org/10.1136/bmjopen-2019-031957

Hansen, B. T., Nygård, M., Castle, P. E., Burger, E. A., & Aasbø, G. (2024). Sociodemographic characteristics associated with cervical cancer screening participation by send-to-all and opt-in HPV self-sampling: Who benefits? Results from a randomized controlled trial among long-term non-attending women in Norway. *Int J Cancer*, 155(6), 1053-1067. https://doi.org/10.1002/ijc.34989

HPV Information Centre. (2023). *Human Papillomavirus and Related Diseases Report Thailand*. I. I. H. I. Centre. https://hpvcentre.net/statistics/reports/THA.pdf?t=1722518262397

Huchko, M. J., Ibrahim, S., Blat, C., Cohen, C. R., Smith, J. S., Hiatt, R. A., & Bukusi, E. (2018). Cervical cancer screening through human papillomavirus testing in community health campaigns versus health facilities in rural western Kenya. *Int J Gynaecol Obstet*, 141(1), 63-69. https://doi.org/10.1002/ijgo.12415

Ibáñez, R., Roura, E., Acera, A., Andújar, M., Pavón, M., Bruni, L., & de Sanjosé, S. (2023). HPV self-sampling among cervical cancer screening users in Spain: A randomized clinical trial of on-site training to increase the acceptability. *Prev Med*, 173, 107571. https://doi.org/10.1016/j.ypmed.2023.107571

Khoo, S. P., Lim, W. T., Rajasuriar, R., Nasir, N. H., Gravitt, P., & Woo, Y. L. (2021). The Acceptability and Preference of Vaginal Self-sampling for Human Papillomavirus (HPV) Testing among a Multi-ethnic Asian Female Population. *Cancer Prev Res (Phila)*, 14(1), 105-112. https://doi.org/10.1158/1940-6207.Capr-20-0280

Lordelo, M. V., Oliveira, C. Z., Aguirre Buexm, L., Vieira Reis, R. M., Longatto-Filho, A., Possati-Resende, J. C., Vazquez, F. L., & Fregnani, J. (2024). Randomized experimental population-based study to evaluate the acceptance and completion of and preferences for cervical cancer screening. *PLoS One*, 19(8), e0306130. https://doi.org/10.1371/journal.pone.0306130

Morgan, K., Azzani, M., Khaing, S. L., Wong, Y. L., & Su, T. T. (2019). Acceptability of Women Self-Sampling versus Clinician-Collected Samples for HPV DNA Testing: A Systematic Review. *J Low Genit Tract Dis*, 23(3), 193-199. https://doi.org/10.1097/lgt.0000000000000476

Nelson, E. J., Maynard, B. R., Loux, T., Fatla, J., Gordon, R., & Arnold, L. D. (2017). The acceptability of self-sampled screening for HPV DNA: a systematic review and meta-

analysis. *Sex Transm Infect*, 93(1), 56-61. https://doi.org/10.1136/sextrans-2016-052609

Nilyanimit, P., Chaithongwongwatthana, S., Oranratanaphan, S., Poudyal, N., Excler, J. L., Lynch, J., Vongpunsawad, S., & Poovorawan, Y. (2024). Comparable detection of HPV using real-time PCR in paired cervical samples and concentrated first-stream urine collected with Colli-Pee device. *Diagn Microbiol Infect Dis*, 108(3), 116160. https://doi.org/10.1016/j.diagmicrobio.2023.116160

Okunade, K. S. (2020). Human papillomavirus and cervical cancer. *J Obstet Gynaecol*, 40(5), 602-608. https://doi.org/10.1080/01443615.2019.1634030

Phoolcharoen, N., Kantathavorn, N., Krisorakun, W., Sricharunrat, T., Teerayathanakul, N., Taepisitpong, C., Sornsamdang, G., Krongthong, W., & Saeloo, S. (2018). Agreement of self- and physician-collected samples for detection of high-risk human papillomavirus infections in women attending a colposcopy clinic in Thailand. *BMC Res Notes*, 11(1), 136. https://doi.org/10.1186/s13104-018-3241-9

Ploysawang, P., Pitakkarnkul, S., Kolaka, W., Ratanasrithong, P., Khomphaiboonkij, U., Tipmed, C., Seeda, K., Pangmuang, P., & Sangrajrang, S. (2023). Acceptability and Preference for Human Papilloma Virus Self-Sampling among Thai Women Attending National Cancer Institute. *Asian Pac J Cancer Prev*, 24(2), 607-612. https://doi.org/10.31557/apjcp.2023.24.2.607

Sangrajrang, S., Pitakkarnkul, S., Muwonge, R., Ploysawang, P., Pangmuang, P., Seeda, K., & Basu, P. (2023). Agreement between Self- and Physician-Sampling for Detection of High-Risk Human Papillomavirus Infections in Women Attending Cervical Screening at National Cancer Institute, Thailand. *Asian Pac J Cancer Prev*, 24(8), 2615-2619. https://doi.org/10.31557/apjcp.2023.24.8.2615

Song, J., Ni, Y. H., Fang, J., Qu, S. X., Chen, X. Y., Wu, W. L., Zhang, W. C., & Qin, J. F. (2024). The levels of women's awareness, experience, acceptability and preference for Vaginal Human Papillomavirus (HPV) self-sampling in three provinces of China: a cross-sectional study. *BMC Womens Health*, 24(1), 343. https://doi.org/10.1186/s12905-024-03186-w

Tranberg, M., Bech, B. H., Blaakær, J., Jensen, J. S., Svanholm, H., & Andersen, B. (2018). Preventing cervical cancer using HPV self-sampling: direct mailing of test-kits increases screening participation more than timely opt-in procedures - a randomized controlled trial. *BMC Cancer*, 18(1), 273. https://doi.org/10.1186/s12885-018-4165-4

Vega-Crespo, B., Neira, V. A., Maldonado-Rengel, R., López, D., Delgado-López, D., Guerra Astudillo, G., & Verhoeven, V. (2024). "Barriers and Advantages of Self-Sampling Tests, for HPV Diagnosis: A Qualitative Field Experience Before Implementation in a Rural Community in Ecuador". *Int J Womens Health*, 16, 947-960. https://doi.org/10.2147/ijwh.S455118

Viveros-Carreño, D., Fernandes, A., & Pareja, R. (2023). Updates on cervical cancer prevention. *Int J Gynecol Cancer*, 33(3), 394-402. https://doi.org/10.1136/ijgc-2022-003703

Viviano, M., Catarino, R., Jeannot, E., Boulvain, M., Malinverno, M. U., Vassilakos, P., & Petignat, P. (2017). Self-sampling to improve cervical cancer screening coverage in Switzerland: a randomised controlled trial. *Br J Cancer*, 116(11), 1382-1388. https://doi.org/10.1038/bjc.2017.111

Winer, R. L., Lin, J., Anderson, M. L., Tiro, J. A., Green, B. B., Gao, H., Meenan, R. T., Hansen, K., Sparks, A., & Buist, D. S. M. (2023). Strategies to Increase Cervical Cancer Screening With Mailed Human Papillomavirus Self-Sampling Kits: A Randomized Clinical Trial. *JAMA*, 330(20), 1971-1981. https://doi.org/10.1001/jama.2023.21471

Sararut Chuleerat. (2024). Development of an educational program on cervical cancer screening using HPV self-sampling among at-risk women at Udon Thani Cancer Hospital. Journal of Health Promotion and Environmental Health, Regional Health Promotion Center 9, 18(1), 351–365. https://he02.tci-thaijo.org/index.php/RHPC9Journal/article/view/266250
