# Supplementary material for: Incremental impact of community-delivered HPV self-sampling on screening uptake within an active outreach system: A quasi-experimental implementation study in rural Thailand
Source: PLoS One. 2026 Jun 1;21(6):e0349531. doi: 10.1371/journal.pone.0349531 (PMC13225424; doi:10.1371/journal.pone.0349531)
Supplement: S3 File — (DOCX) [file pone.0349531.s003.docx]

**S3 Table. Reasons for not participating in sample collection among non-participants.**

| **Reasons** | **Community-delivered with active outreach**  **group (n=26)** | | **Facility-based with active outreach group**  **(n=34)** | | **P-value*** |
| --- | --- | --- | --- | --- | --- |
|  | **n** | **%** | **n** | **%** |  |
| Lack of time | 17 | 65.38 | 25 | 73.53 | 0.16 |
| Work obligations | 8 | 30.77 | 6 | 17.65 |  |
| Collected samples independently | 1 | 3.84 | 0 | 0 |  |
| Fear of the results | 0 | 0 | 3 | 8.82 |  |

** Based on the Chi-square testing*
