# Supplementary material for: Incremental impact of community-delivered HPV self-sampling on screening uptake within an active outreach system: A quasi-experimental implementation study in rural Thailand
Source: PLoS One. 2026 Jun 1;21(6):e0349531. doi: 10.1371/journal.pone.0349531 (PMC13225424; doi:10.1371/journal.pone.0349531)
Supplement: S4 File — (DOCX) [file pone.0349531.s004.docx]

**S4 Table. Self-sampling experience between study arms (n = 46).**

| **Self-sampling experience** | **Community-delivered with active outreach**  **group (n=28)** | | **Facility-based with active outreach group (n=18)** | | **P-value*** |
| --- | --- | --- | --- | --- | --- |
|  | n | % | n | % |  |
| **1.** **What do you think of this experience?** | | | | | |
| Very good | 4 | 14.29 | 5 | 27.78 | 0.27 |
| Good | 18 | 64.29 | 10 | 55.56 |  |
| Neither good nor bad | 4 | 14.29 | 1 | 5.55 |  |
| Bad | 0 | 0.00 | 1 | 5.55 |  |
| Very bad | 0 | 0.00 | 0 | 0.00 |  |
| I don’t know | 2 | 7.14 | 1 | 5.55 |  |
| **2.** **Was it easy for you to understand the instructions to perform the self-sample?** | | | | | |
| Yes, they were very clear and easy to understand | 20 | 71.43 | 16 | 88.89 | 0.26 |
| Quite clear and easy to understand | 5 | 17.86 | 2 | 11.11 |  |
| Normal, not so simple, or so complicated | 3 | 10.71 | 0 | 0.00 |  |
| It was a bit difficult for me to understand | 0 | 0.00 | 0 | 0.00 |  |
| It was very difficult for me to understand the instructions | 0 | 0.00 | 0 | 0.00 |  |
| Other: | 0 | 0.00 | 0 | 0.00 |  |
| I don’t know/I prefer not to answer | 0 | 0.00 | 0 | 0.00 |  |
| **3.** **Was the brush/swab easy to introduce?** | | | | | |
| Yes, it goes in easily without problems | 21 | 75.00 | 16 | 88.89 | 0.049 |
| Yes, but it’s a little difficult | 5 | 17.86 | 2 | 11.11 |  |
| Normal / indifferent | 2 | 7.14 | 0 | 0.00 |  |
| It took me a while to introduce it | 0 | 0.00 | 0 | 0.00 |  |
| It was very hard to introduce it | 0 | 0.00 | 0 | 0.00 |  |
| Other: | 0 | 0.00 | 0 | 0.00 |  |
| I don’t know/I prefer not to answer | 0 | 0.00 | 0 | 0.00 |  |
| **4. What do you think about the use of the self-sample kit?** | | | | | |
| I thought it was very easy and simple to use | 21 | 75.00 | 17 | 94.44 | 0.29 |
| I thought it was easy to use | 2 | 7.14 | 1 | 5.55 |  |
| Normal, not difficult nor easy | 4 | 14.29 | 0 | 0.00 |  |
| I thought it was a little complicated to use | 1 | 3.57 | 0 | 0.00 |  |
| I thought it was very complicated to use | 0 | 0.00 | 0 | 0.00 |  |
| I don’t know/I prefer not to answer | 0 | 0.00 | 0 | 0.00 |  |
| **5. Do you think you collected the sample properly?** | | | | | |
| Yes, I’m sure | 22 | 78.57 | 16 | 88.89 | 0.17 |
| Yes, but I have some doubts | 6 | 21.43 | 1 | 5.55 |  |
| I’m not sure that I collected the sample properly | 0 | 0.00 | 1 | 5.55 |  |
| I’m sure I did not collect the sample properly | 0 | 0.00 | 0 | 0.00 |  |
| Other: | 0 | 0.00 | 0 | 0.00 |  |
| I don’t know/I prefer not to answer | 0 | 0.00 | 0 | 0.00 |  |
| **6. Did you feel any pain when you were introducing the brush?** | | | | | |
| No, I did not feel any pain | 17 | 60.7 | 9 | 50.00 | 0.49 |
| No, but I felt some discomfort | 4 | 14.29 | 2 | 11.11 |  |
| Yes, a little intense | 7 | 25.00 | 5 | 27.78 |  |
| Yes, an intense pain | 0 | 0.00 | 1 | 5.55 |  |
| Other: | 0 | 0.00 | 1 | 5.55 |  |
| I don’t know/I prefer not to answer | 0 | 0.00 | 0 | 0.00 |  |
| **7. What else did you feel when you were performing the self-sampling?** | | | | | |
| **Comfort** |  |  |  |  |  |
| None | 1 | 3.57 | 0 | 0.00 | 0.33 |
| Little | 0 | 0.00 | 1 | 5.55 |  |
| Normal | 10 | 35.71 | 3 | 16.67 |  |
| Quite | 3 | 10.71 | 4 | 22.22 |  |
| A lot | 14 | 50.00 | 10 | 55.56 |  |
| I don’t know | 0 | 0.00 | 0 | 0.00 |  |
| **Calmness** |  |  |  |  |  |
| None | 1 | 3.57 | 1 | 5.55 | 0.74 |
| Little | 1 | 3.57 | 0 | 0.00 |  |
| Normal | 7 | 25.00 | 3 | 16.67 |  |
| Quite | 5 | 17.86 | 2 | 11.11 |  |
| A lot | 14 | 50.00 | 12 | 66.67 |  |
| I don’t know | 0 | 0.00 | 0 | 0.00 |  |
| **Normality** |  |  |  |  |  |
| None | 0 | 0.00 | 1 | 5.55 | 0.12 |
| Little | 0 | 0.00 | 0 | 0.00 |  |
| Normal | 5 | 17.86 | 0 | 0.00 |  |
| Quite | 7 | 25.00 | 3 | 16.67 |  |
| A lot | 16 | 57.14 | 14 | 77.78 |  |
| I don’t know | 0 | 0.00 | 0 | 0.00 |  |
| **Safety** |  |  |  |  |  |
| None | 0 | 0.00 | 0 | 0.00 | 0.21 |
| Little | 1 | 3.57 | 0 | 0.00 |  |
| Normal | 3 | 10.71 | 1 | 5.55 |  |
| Quite | 7 | 25.00 | 1 | 5.55 |  |
| A lot | 17 | 60.71 | 16 | 88.89 |  |
| I don’t know | 0 | 0.00 | 0 | 0.00 |  |
| **Privacy** |  |  |  |  |  |
| None | 0 | 0.00 | 0 | 0.00 | 0.22 |
| Little | 2 | 7.14 | 0 | 0.00 |  |
| Normal | 0 | 0.00 | 0 | 0.00 |  |
| Quite | 5 | 17.86 | 1 | 5.55 |  |
| A lot | 21 | 75.00 | 17 | 94.44 |  |
| I don’t know | 0 | 0.00 | 0 | 0.00 |  |
| **Shame** |  |  |  |  |  |
| None | 22 | 78.6 | 15 | 83.33 | 0.32 |
| Little | 1 | 3.57 | 2 | 11.11 |  |
| Normal | 5 | 17.86 | 1 | 5.55 |  |
| Quite | 0 | 0.00 | 0 | 0.00 |  |
| A lot | 0 | 0.00 | 0 | 0.00 |  |
| I don’t know | 0 | 0.00 | 0 | 0.00 |  |
| **Fear** |  |  |  |  |  |
| None | 21 | 75.00 | 15 | 83.33 | 0.77 |
| Little | 3 | 10.71 | 1 | 5.55 |  |
| Normal | 4 | 14.29 | 2 | 11.11 |  |
| Quite | 0 | 0.00 | 0 | 0.00 |  |
| A lot | 0 | 0.00 | 0 | 0.00 |  |
| I don’t know | 0 | 0.00 | 0 | 0.00 |  |
| **Anxiety** |  |  |  |  |  |
| None | 21 | 75.00 | 16 | 88.89 | 0.51 |
| Little | 3 | 10.71 | 1 | 5.55 |  |
| Normal | 4 | 14.29 | 1 | 5.55 |  |
| Quite | 0 | 0.00 | 0 | 0.00 |  |
| A lot | 0 | 0.00 | 0 | 0.00 |  |
| I don’t know | 0 | 0.00 | 0 | 0.00 |  |
| **Frustration** |  |  |  |  |  |
| None | 26 | 92.86 | 17 | 94.44 | 0.69 |
| Little | 1 | 3.57 | 1 | 5.55 |  |
| Normal | 1 | 3.57 | 0 | 0.00 |  |
| Quite | 0 | 0.00 | 0 | 0.00 |  |
| A lot | 0 | 0.00 | 0 | 0.00 |  |
| I don’t know | 0 | 0.00 | 0 | 0.00 |  |
| **Nervousness** |  |  |  |  |  |
| None | 23 | 82.14 | 15 | 83.33 | 0.24 |
| Little | 2 | 7.14 | 3 | 16.67 |  |
| Normal | 3 | 10.71 | 0 | 0.00 |  |
| Quite | 0 | 0.00 | 0 | 0.00 |  |
| A lot | 0 | 0.00 | 0 | 0.00 |  |
| I don’t know | 0 | 0.00 | 0 | 0.00 |  |
| **8.** **How long did it take to collect the gynecological sample?** | | | | | |
| Less than 5 minutes | 17 | 60.71 | 17 | 94.44 | 0.038 |
| Between 6 and 10 minutes | 9 | 32.14 | 1 | 5.55 |  |
| Between 11 and 15 minutes | 2 | 7.14 | 0 | 0.00 |  |
| Between 16 and 20 minutes | 0 | 0.00 | 0 | 0.00 |  |
| Between 20 and 25 minutes | 0 | 0.00 | 0 | 0.00 |  |
| More than 25 minutes | 0 | 0.00 | 0 | 0.00 |  |
| Mean ± SD | 5.75±1.55 | | 5.06±0.24 | | 0.07 |
| **9.** **Did you get any help collecting the sample?** | | | | | |
| Yes, but only to understand the instructions | 5 | 17.86 | 5 | 27.78 | 0.69 |
| Yes, to understand the instructions and to collect the sample | 6 | 21.43 | 4 | 22.22 |  |
| No, I did it myself | 17 | 60.71 | 9 | 50.00 |  |
| Other | 0 | 0.00 | 0 | 0.00 |  |
| I don’t know/I prefer not to answer | 0 | 0.00 | 0 | 0.00 |  |
| **10.** **Do you think the self-sample will not cause you any harm?** | | | | | |
| Yes | 18 | 64.29 | 10 | 55.56 | 0.55 |
| No | 10 | 35.71 | 8 | 44.44 |  |
| Other | 0 | 0.00 | 0 | 0.00 |  |
| I don’t know/I prefer not to answer | 0 | 0.00 | 0 | 0.00 |  |
| **11.** **Do you trust the test results?** | | | | | |
| Yes | 25 | 89.29 | 15 | 83.33 | 0.63 |
| No | 2 | 7.14 | 1 | 5.55 |  |
| Other | 1 | 3.57 | 0 | 0.00 |  |
| I don’t know/I prefer not to answer | 0 | 0.00 | 2 | 11.11 |  |
| **12. Do you think that this test benefits your health?** | | | | | |
| Yes | 27 | 96.43 | 16 | 88.89 | 0.28 |
| No | 1 | 3.57 | 0 | 0.00 |  |
| Other | 0 | 0.00 | 1 | 5.55 |  |
| I don’t know/I prefer not to answer | 0 | 0.00 | 1 | 5.55 |  |
| **13.** **Would you recommend this test to a friend or family member?** | | | | | |
| Yes | 28 | 100.00 | 18 | 100.00 |  |
| No | 0 | 0.00 | 0 | 0.00 |  |
| Other | 0 | 0.00 | 0 | 0.00 |  |
| I don’t know/I prefer not to answer | 0 | 0.00 | 0 | 0.00 |  |
| **14.** **What do you prefer, self-sampling (to collect the sample yourself) or to have a health professional collect the sample for cervical cancer screening?** | | | | | |
| Myself | 17 | 60.71 | 14 | 77.78 | 0.39 |
| Healthcare professional | 5 | 17.86 | 1 | 5.55 |  |
| I’m OK with both options | 6 | 21.43 | 3 | 16.67 |  |
| None of the options | 0 | 0.00 | 0 | 0.00 |  |
| Other | 0 | 0.00 | 0 | 0.00 |  |
| I don’t know/I prefer not to answer | 0 | 0.00 | 0 | 0.00 |  |
| **15.** **Would you like to see self-sampling kits as a screening approach in the future?** | | | | | |
| Yes | 25 | 89.29 | 17 | 94.44 | 0.49 |
| No | 1 | 3.57 | 1 | 5.55 |  |
| Other | 2 | 7.14 | 0 | 0.00 |  |
| I don’t know/I prefer not to answer | 0 | 0.00 | 0 | 0.00 |  |
| **16.** **If it was to be used in the future, where would like to pick up/find the self-sampling kit?** | | | | | |
| At the primary health service centre | 16 | 57.14 | 13 | 72.22 | 0.33 |
| At the gynaecological centre | 1 | 3.57 | 2 | 11.11 |  |
| At a pharmacy | 0 | 0.00 | 0 | 0.00 |  |
| At a post office or mailbox | 1 | 3.57 | 0 | 0.00 |  |
| To be shipped to my house | 10 | 35.71 | 3 | 16.67 |  |
| Other | 0 | 0.00 | 0 | 0.00 |  |
| I don’t know/I prefer not to answer | 0 | 0.00 | 0 | 0.00 |  |
| **17.** **Where would you like to return to the self-sampling kit once the sample is collected?** | | | | | |
| At the primary health service centre | 17 | 60.71 | 13 | 72.22 | 0.25 |
| At the gynaecological centre | 0 | 0.00 | 1 | 5.55 |  |
| At a pharmacy | 0 | 0.00 | 0 | 0.00 |  |
| At a post office or mailbox | 0 | 0.00 | 0 | 0.00 |  |
| To be shipped to my house | 11 | 39.29 | 4 | 22.22 |  |
| Other | 0 | 0.00 | 0 | 0.00 |  |
| I don’t know/I prefer not to answer | 0 | 0.00 | 0 | 0.00 |  |

*^*^ Based on the Chi-square testing*
